# Proline Residues as Switches in Conformational Changes Leading to Amyloid Fibril Formation

**DOI:** 10.3390/ijms18030549

**Published:** 2017-03-07

**Authors:** Ajda Taler-Verčič, Samra Hasanbašić, Selma Berbić, Veronika Stoka, Dušan Turk, Eva Žerovnik

**Affiliations:** 1 Department of Biochemistry and Molecular and Structural Biology, Jožef Stefan Institute, Jamova 39, 1000 Ljubljana, Slovenia; ajda.taler@ijs.si (A.T.-V.); veronika.stoka@ijs.si (V.S.); dusan.turk@ijs.si (D.T.); 2 CIPKeBiP—Center of Excellence for Integrated Approaches in Chemistry and Biology of Proteins, Jamova 39, 1000 Ljubljana, Slovenia; 3 Jožef Stefan International Postgraduate School, Jamova 39, 1000 Ljubljana, Slovenia; samra1988@hotmail.com; 4 Faculty of Pharmacy, Department of Biochemistry, University of Tuzla, Univerzitetska 1, 75000 Tuzla, Bosnia and Herzegovina; selma.berbic@untz.ba

**Keywords:** *cis* proline, conformational switch, folding intermediate, domain swapping, amyloid fibrils, protein aggregation, stefin B, β_2_-microglobulin

## Abstract

Here we discuss studies of the structure, folding, oligomerization and amyloid fibril formation of several proline mutants of human stefin B, which is a protein inhibitor of lysosomal cysteine cathepsins and a member of the cystatin family. The structurally important prolines in stefin B are responsible for the slow folding phases and facilitate domain swapping (Pro 74) and loop swapping (Pro 79). Moreover, our findings are compared to β_2_-microglobulin, a protein involved in dialysis-related amyloidosis. The assessment of the contribution of proline residues to the process of amyloid fibril formation may shed new light on the critical molecular events involved in conformational disorders.

## 1. Introduction

**Figure 1:**
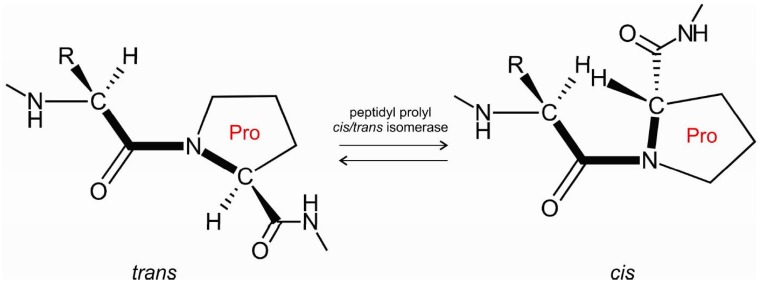
Peptidyl-prolyl *cis*/*trans* isomerase facilitates *cis*/*trans* isomerization of the X-Pro peptide bond.

Proline residues play a prominent role in protein folding [1,2], protein mis-folding, and aggregation [3]. They are key to attaining the functional state of proteins [4]. Prolines also play a role in domain-swapping [5,6] and in protein aggregation to amyloid fibrils [7,8,9]. Peptidyl-prolyl *cis*/*trans* isomerases are enzymes that catalyze the *cis*/*trans* isomerization of peptide bonds preceding prolines (Figure 1). The *cis*/*trans* isomerization of the peptide bond acts as a molecular switch controlling several physiologically important processes, such as opening of the pore of a neurotransmitter-gated ion channel [10] or the formation of α-synuclein inclusions [11] in Parkinson’s disease.

Amyloid fibril formation is a generic property among most proteins [12,13]. The cystatins, which are protein inhibitors of lysosomal cysteine cathepsins, are a model for studies of amyloid fibril formation. The cystatin family consists of three types of inhibitory proteins, namely, stefins (type-1), cystatins (type-2) and kininogens (type-3). Stefins are intracellular proteins present in the cytosol [14,15], including stefins A and B in humans [16] and stefins, A, B and C in bovidae [17,18]. Human stefin B [19,20,21,22,23], chimeric stefins [24] and cystatin C [25,26] have been used as suitable model proteins to study protein folding and amyloid fibril formation. Human stefin B is a small globular protein consisting of 98 amino acids with no disulfide bonds; its native sequence possesses a free Cys residue at position 3. To avoid intermolecular disulfide bridge formation, this Cys is changed into Ser for all in vitro studies (hereafter referred to wild-type protein, wt). This cytoplasmic protein is supposed to act primarily as a cysteine protease inhibitor [15], scavenging and inhibiting accidentally released cathepsins from the lysosome. In addition, stefin B also resides in the nucleus [27] where a number of alternative functions have been proposed. Stefin B (also termed cystatin B) gene mutations, either dodecamer repeats resulting in lower protein production or missense mutations leading to misfolding, cause a progressive myoclonus epilepsy of type 1 (EPM1) with slow signs of neurodegeneration [28,29]. Similarly to cystatin C, stefin B protects neurons from excessive oxidative stress [30,31] and protein misfolding [32]. Alternative functions, such as amateur chaperone function, have also been suggested from both experimental data and bioinformatic analysis [33]. A breakthrough in the understanding of the structure of cystatins and their mechanism of interaction with papain-like cysteine proteases, including lysosomal cathepsins, was provided by the three-dimensional (3D) structures of chicken cystatin monomer [34,35] and human stefin B-papain complex [36].

Our in vitro studies of stefin B folding revealed several slow phases [37,38], which were accompanied by dimerization of the protein. We were able to determine the crystal structure of a stefin B tetramer, which is composed of two domain-swapped dimers [19]. Of note, in stefin B, the proline residue at position 74 in the tetramer is in a *cis* conformation [19]. These structures were crucial for further development in the study of proteolysis and its inhibition, and represent the basis for understanding the mechanism of amyloid-fibril formation through 3D-domain swapping.

The *cis*-to-*trans* proline isomerization [2] is a slow process, dependent on pH. Stefin B has in total five proline residues at positions 6, 11, 36, 74 and 79. We have examined in more detail prolines at positions 74 and 79, and both have proved to be structurally relevant. When Pro 79 was mutated into a Ser in a stefin B-Y31 variant (with Y at site 31), the protein oligomerized predominantly as a tetramer which could be crystallized [19]. If Pro 74 was mutated into a Ser in the same variant, it underwent a transition to an oligomeric molten globule state [19,39]. We also studied the stefin B-Y31 P36G mutant, which rendered the protein less stable [40]. Historically, while the stefin B-Y31 variant [41] was observed and characterized first, the E31 variant is now referred to as wild-type since it is the most abundant.

To put our work in a wider context, we describe another protein where prolines dictate folding and amyloid fibril formation, β_2_-microglobulin (β_2_m). Interestingly, β_2_m shares with stefin B the same number of prolines, at positions 5, 14, 32, 72 and 90, and we compare the two systems in our Discussion and Conclusions sections.

## 2. Results

### 2.1. Influence of Prolines on Folding and Stability of Stefin B

In the early folding studies using a stefin B-Y31 variant, we observed that the protein, in contrast to stefin A, undergoes slow folding phases that are a repetition of the fast folding phases [37]. The amplitude of the slow phases is about 25%–30%. This can be explained by the existence of a population of molecules in the denatured state with either one or two non-native (*cis*) proline isomers that undergo similar, albeit slow, folding as the fast folding molecules with native (*trans*) proline configuration in the denatured state. However, the final oligomeric state of the slow phase proves to be dimeric, thus the dimer can stabilize a structurally important proline in a *trans* conformation. If a proline were *cis*, one would expect a higher amplitude of the slow phases, amounting to 70%, not only 30%. When we used size exclusion chromatography (SEC) to study the Y31 variant and its P36G mutant, we showed that 70% and 75% of molecules were monomers, respectively and the rest were dimers, whereas for the P79S mutant of the same variant, 100% were dimeric. This points to Pro 79 *trans* to *cis* isomerization as the very likely cause for the slow phase of folding towards a dimer. Nevertheless, in the tetrameric structure of the stefin B-Y31 P79S mutant, two domain-swapped dimers form the tetramer in which the Pro 74 was found in *cis* conformation [19].

### 2.2. Influence of Prolines on Conformation and Oligomerization of Stefin B

**Figure 2:**
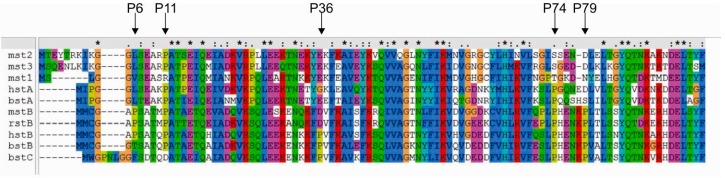
Sequence alignment of different stefins. Mouse stefin B (mstB), human stefin A (hstA), rat stefin B (rstB), mouse stefin 1 (mst1), mouse stefin 2 (mst2), mouse stefin 3 (mst3), bovine stefin A (bstA), bovine stefin B (bstB) and bovine stefin C (bstC) were compared against human stefin B (hstB). Sequences were retrieved from UniProt database. The multiple sequence alignment was performed with ClustalX [42]. All five proline residues (P6, P11, P36, P74 and P79) are indicated with an arrow.

As we observed that the wt stefin B (as defined in the Introduction) is more stable and less prone to forming a molten globule, we studied the role of all five single-point proline mutants of stefin B. Using multiple sequence alignment of several stefins, we identified common amino acid substitution of the prolines in human stefin B (Figure 2). The five prolines at positions 6, 11, 36, 74 and 79 were mutated to leucine, serine, aspartic acid, serine and serine, respectively. All mutant proteins were produced in an *Escherichia coli (E. coli)* expression system (Supplementary Figure S1) and were shown to retain their inhibitory activity (Supplemenatry Figure S2).

**Figure 3:**
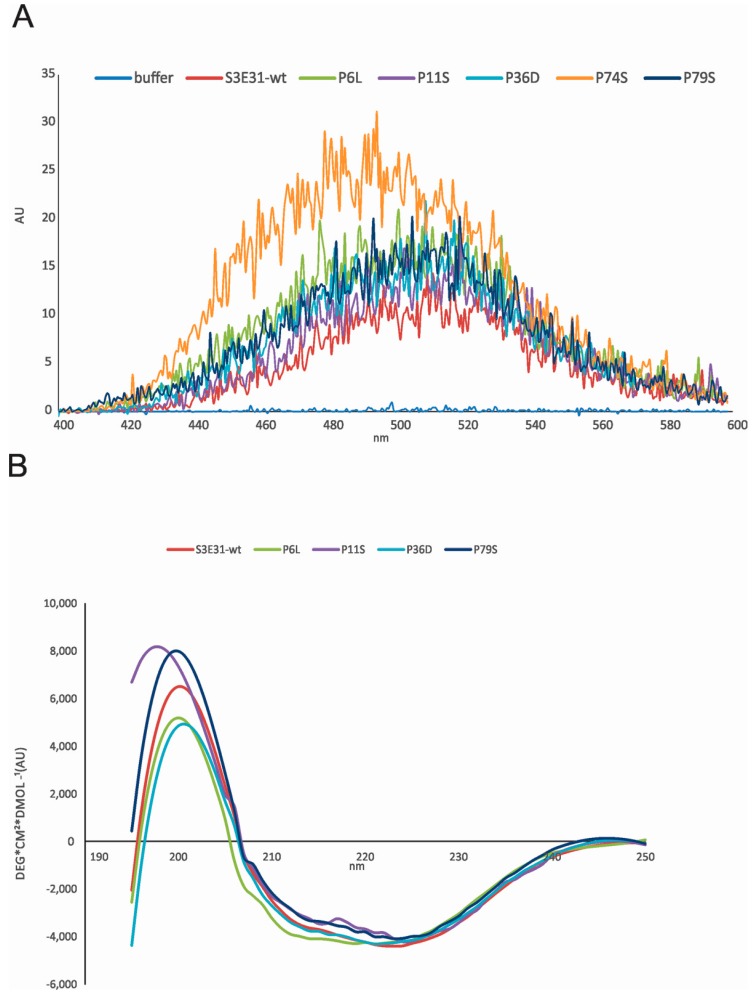
(**A**) ANS fluorescence upon binding to wild-type protein (wt) stefin B and proline mutants. The excitation wavelength was 370 nm, and the spectra were recorded from 400 nm to 600 nm on a Perkin Elmer LS50B (Perkin Elmer, Waltham, MA, USA). Slits were open 2.5 nm. ANS was dissolved in 0.01 M phosphate buffer, pH 7, and 0.15 M NaCl. Final concentrations were 1.25 mM ANS and 25 µM proteins; (**B**) Far UV CD spectra of wt stefin B and proline mutants were recorded from 195 to 250 nm, as indicated. The bandwidth was 1 nm and spectra recording time at each nm was 3 s; the temperature was 25 °C. Final concentrations were 34 µM. Due to high aggregation propensity, the final concentrations varied. Therefore, ellipticity values were normalized to the wt spectrum (i.e., a factor was used to give ellipticity—4200 ± 100 deg·cm^2^·dmol^−1^).

The stability and exposure of hydrophobic patches were confirmed by measuring ANS (1-anilinonaphthalene-8-sulfonic acid) fluorescence spectra (Figure 3A). Together with far UV-CD (ultraviolet circular dichroism) spectra (Figure 3B) we observed that P6L, P11S, P36D and P79S have hydrophobic exposure and secondary structure similar to the wild-type (wt) protein. The highest ANS binding is observed for the P74S mutant indicating a molten globule-like state. However, this was not observed consistently for the P74S mutant of the wt [20] by measuring CD spectra. It may well be that the molten globule intermediate forms only under destabilizing conditions or upon freeze-thawing cycles.

SEC data on the wt and its proline mutants show (Supplementary Figure S3) that dimers are the main oligomeric form of the wt stefin B and P36D mutant—when frozen and unfrozen once. The amount of the higher oligomers increases upon freeze-thawing. Then come the tetramers and higher oligomers. An estimate from the surface area of the peaks indicates 65% dimers, 20% tetramers, 5% monomers and 10% of higher oligomers. A similar distribution of the oligomers was obtained for P6L and P11S of the wt stefin B. In the case of P79S, the tetramer peak amounted to a higher percentage of around 30% tetramers, 55% dimers and 15% of higher molecular weight species. The tendency to form oligomers is high for the P74S mutant of the wt (>50%), in accordance with its tendency to transform into an oligomeric molten globule as observed previously for the Y31 variant [19]. The P79S mutant of the the Y31 variant was predominantly in the form of tetramers [19]. Taken together, stabilization of the dimer of stefin B is sensitive to Pro 36, whereas stabilization of the tetramer is sensitive to Pro 79. When *cis* to *trans* transition is facilitated by the Pro mutation at the two sites, respectively, dimers and tetramers are populated to a higher amount.

### 2.3. Prediction of the Effects of Proline Mutations on Human Stefin B Stability

The prediction of human stefin B stability upon single-point mutations of proline residues in the protein sequences (UniProt ID: P04080 and its mutant C3S) [43] as well as the monomeric (1STF:I [36] and 4N6V:chain0 [44] and tetrameric 2OCT:chainA [19]) protein structures, was performed at pH 7.0 and 25 °C using a support vector machine (SVM)-based tool, I-Mutant2.0 [45]. Of note, the protein stability increased only for the P6L mutant, whereas all remaining mutations, namely P36D and three Pro to Ser mutations at positions 11, 74 and 79, decreased protein stability (Table 1).

### 2.4. Influence of Prolines on Amyloid Fibril Formation of Human Stefin B

Substituting proline at position 74 with a serine in the sequence of the wt stefin B did not affect the protein structure and stability to any significant extent, as shown by urea and thermal denaturation [20]. In fact, the mutant was slightly more stable, which is in contrast to the prediction in Table 1 (one, however, has to bear in mind that the changes in stability in both cases: prediction and experiment, are rather small and within the standard error of 1.4 ± 0.1 kcal/mol of the I-Mutant2.0 program). The exchange of a proline would be expected to lead to a more stable protein, due to higher flexibility—i.e., entropic contribution to stability, however, enthalpic contribution and hydration effects increase or decrease the stability.

**Figure 4:**
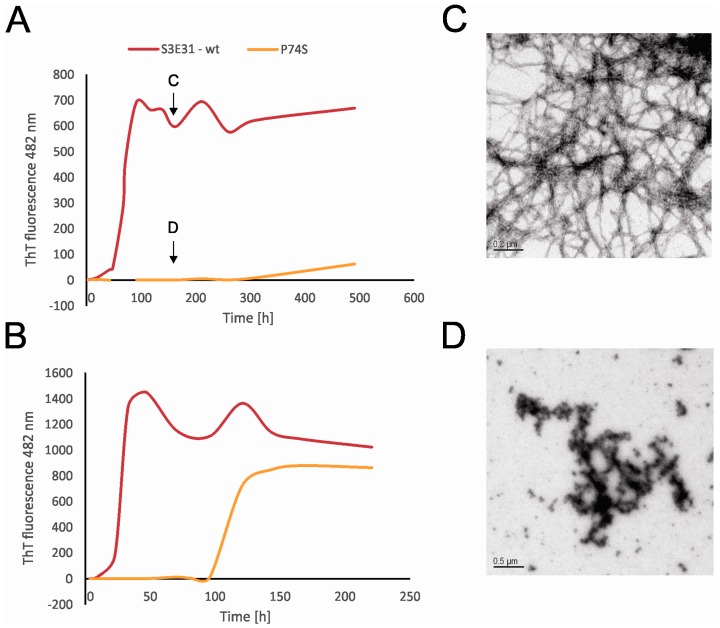
The time courses of amyloid fibril formation of wild-type stefin B and its site-specific mutant P74S were monitored by ThT fluorescence at 482 nm. The fibrillation reactions took place in 0.015 M acetate buffer, 0.15 M NaCl, pH 4.8, 12% TFE at 25 °C (**A**) and in the same buffer at 30 °C (**B**). TEM images taken during the fibrillation reactions. The wild-type stefin B (**C**) and P74S mutant (**D**) on the 7th day of fibrillation (see arrows) [20].

When the fibrillation rate of the P74S mutant was compared to the fibrillation rate of the wt-like protein, however, it was shown that P74 is essential not only for stefin B tetramer formation but also for amyloid fibrillation. Indeed, when Pro74 was replaced with Ser, the lag phase was extended up to 10 times with a smaller final yield (Figure 4A,B). CD spectra show that this mutant adopts a folded structure, thus these differences are not the result of a change in the overall fold of the mutant. Transmission electron microscopy (TEM) results (Figure 4C,D) reveals that P74S remains in the form of granular aggregates (Figure 4D), whereas the wt protein formed amyloid fibrils after 7 days of incubation (Figure 4C). Moreover, when the effects of peptidyl-prolyl isomerase cyclophilin A (CypA) were examined, it was shown that CypA prolongs the lag phase and increases the final yield and length of the fibrils. On the other hand, the inactive cyclophilin A R55A caused a prolonged lag phase, but did not lead to an increase in the final fibril yield [20]. Although the fibrils formed in the presence or absence of CypA had the same shape and morphology, the presence of CypA provides a higher yield of stefin B fibrils [20].

### 2.5. Structure of Monomer and Tetramer Composed of Domain-Swapped Dimers of Stefin B

**Figure 5:**
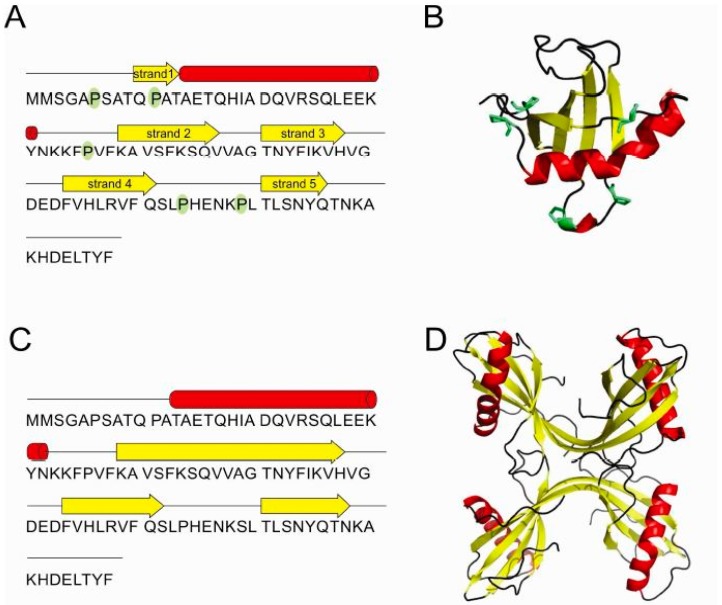
Stefin B—schematic representation of secondary structure elements (**A**) and the 3D structure of monomer (PDB id: 1STF) [36] (**B**). Schematic of secondary structure elements (**C**) of the two domain-swapped dimers building-up the tetramer (PDB id: 2OCT) [19] (**D**). All five prolines are highlighted in green in the monomer.

The crystal structure of the monomer of stefin B (Figure 5A,B) determined in a complex with papain was one of the first structures of cystatins and as such represented a cornerstone in our ability to understand the mechanism of its inhibitory action on proteases of the papain family [36]. The monomer is a typical α/β protein, with a well-formed β-sheet of 5 β-strands and an α-helix (residues 12 to 37). As an interesting point, a monomer of stefin B crystallized at pH 10 [44], showed a 4-dimensional arrangement in the crystal cage, resembling a channel.

The monomeric structure of stefin B [36] was also crucial for further understanding the mechanism of amyloid-fibril formation through 3D-domain swapping. One mechanism for oligomerization is a 3D domain-swapping mechanism [26,46,47] where an intramolecular interface from one monomer becomes an intermolecular interface between subunits in the oligomers [48]. First, the crystal structure of cystatin C domain-swapped dimer was determined [49], closely followed by the NMR-derived structure of stefin A [26]. In the domain-swapped dimer of stefins, each stefin fold is made of strand 1, the α-helix and strand 2 from one monomer and strands 3–5 from the other monomer. In addition, the stefin B tetramer has been shown to have two domain-swapped dimeric units which interact through loop-swapping, also termed “hand-shaking” (Figure 5C,D).

In the case of stefin B it has been demonstrated that prolines play an important role in domain swapping, as they control the rigidity of loops between secondary structure elements. The *trans* conformer of Pro 74 is found widely conserved among stefins and cystatins [26,49], whereas the *cis* conformer is reported in the structure of the stefin B tetramer only [19]. This isomer is particularly important as it brings the Ser 72-Leu 80 loop in the close vicinity of the N-terminal trunk. In the loop swap of two domain-swapped dimer units, the loop position from residues Ser 72 to Leu 80 is provided by Pro 74 and Pro 79. Pro 79 contributes to the rigidity of the loop through its *trans* conformation.

## 3. Discussion

For comparison with our model protein stefin B data, we reviewed the literature on the role of proline isomerization in the structure, folding and amyloid fibril formation of β_2_-microglobulin (β_2_m). Furthermore, we predicted how chosen proline mutations may influence the stability of this protein.

β_2_m is a 99 amino acid long protein containing the light-chain of the major histocompatibility complex I (MHC I) [50]. β_2_m is present at the surface of almost all cells. Upon dissociation from MHC I it is catabolized in the kidneys. Therefore, in patients who suffer from chronic kidney insufficiency and undergo dialysis treatment, the concentration of β_2_m increases up to 60-fold causing dialysis-related amyloidosis (DRA), i.e., insoluble β_2_m amyloids form and accumulate in the joints and in connective tissues [51,52]. Intriguingly, high concentrations of β_2_m cannot completely clarify the onset of amyloid precipitation, as in vitro studies have shown that this protein stays soluble and monomeric at neutral pH even when concentrations are more than 100 times higher than in patients exposed to dialysis [52,53].

### 3.1. Influence of Prolines in β_2_-Microglobulin: Folding and Oligomerization

Chiti et al. [54] have shown that β_2_m folds via two structurally different intermediates on its way to the globular native state. One of these, termed I_1_, is populated within 5 ms and contains a disorganized hydrophobic core with several hydrophobic residues exposed to solvent. The other one, termed I_2_, forms within ms from the I_1_ species and shows a native-like secondary structure with side chains packed in the hydrophobic core. [54] This species further folds to a globular native state within an interval ranging from seconds to minutes at 30 °C. Further studies demonstrated that the slow folding of I_2_ which precedes the native state is rate limited by *trans* to *cis* isomerization of the His 31-Pro 32 peptide bond [55].

β_2_m fibril formation starts rapidly at low pH with lag-dependent kinetics where dimers, trimers and tetramers are formed [56,57]. Studies of the kinetics of fibril formation have shown that monomers form a nucleus consisting of six β_2_m polypeptide chains, whereas fibrils are formed in the elongation phase by adding monomers [56]. Even though several β_2_m oligomeric species have been characterized [58] the linkage between oligomers and fibrils remains unknown.

Oligomerization is considered as a crucial step towards self-association of proteins into amyloid fibrils. Moreover, oligomers are believed to be toxic in several types of amyloid-related neurodegenerative diseases [59,60,61,62]. Exploring the molecular mechanisms leading to the formation of oligomers is a great challenge, as it would help in developing strategies to suppress amyloid-related diseases. Toxicity is not restricted to pathological proteins alone, it is instead related to a common structural/conformational property of the prefibrillar oligomers [61,62]. The mechanism through which β_2_m causes DRA remains poorly understood. It has been reported that β_2_m forms nonselective, long-lived and voltage-independent ion channels in phospholipid bilayers and that their appearance is tightly correlated with DRA [63]. These channels can bind Congo red and zinc, hence it was suggested by the authors that their structure includes β-sheets [63]. On a separate note, it is also not clear whether it takes the full-length protein to develop the pathophysiology or whether fragments can cause it. In order to clarify this issue, Mustata and coworkers designed K3, which is a digestion fragment of the full length β_2_m (Ser 20-Lys 41) [64]. It is known that this peptide forms amyloid fibrils under a wide range of conditions [64]. Combining solid state NMR, atomic force microscopy and X-ray diffraction, the characteristic amyloid conformation was elucidated; thus showing that K3 has adopted a U-shaped β-strand-turn-β-strand motif [64]. Interestingly, this motif had already been reported as a universal amyloid feature and hence it was speculated that it might play a role in toxicity [65,66,67]. Moreover, the same authors have proven by channel modelling that this K3 oligomer can constitute the structure of the channel. These results, together with fluorescence measurements in kidney cells which have shown channel-mediated calcium uptake, indicate that the β_2_m related DRA can be mediated by ion channels formed by the K3 fragment [64]. These data add weight to the so called “channel hypothesis”; these channels lead to Ca^2+^ influx which can cause apoptosis and alter signaling, hence changing the plasma membrane and electrical properties of the neuron.

### 3.2. Aggregation and Amyloid-Fibril Formation of β_2_-Microglobulin

β_2_m has been widely used as a powerful model for exploration of the structural molecular mechanisms of fibril formation from a full-length protein in vitro. Natively, this protein folds into a β-sandwich fold consisting of 2 β-sheets, one containing 4 strands and the other 3, which are covalently linked by a disulphide bond between 2 cysteines (residues 26 and 81) [68]. It contains five peptidyl-prolyl bonds and one of them (His 31-Pro 32) exhibits a thermodynamically unfavorable *cis*-isomer conformation in the native state [52].

A huge body of evidence has shown that ~60% of the sequence of β_2_m is highly amyloidogenic [69,70,71]. Nevertheless, the natively folded protein is not prone to aggregation [72,73] which implies that the folded structure strongly affects its amyloidogenic potential. The partial unfolding in vivo therefore appears to be a mandatory step leading to aggregation as it provides the exposure of aggregation-prone regions of the sequence. β_2_m spontaneously forms fibrils in vitro at pH < 3.0 with low ionic strength (<50 mM NaCl) when stimulated by agitation [57]. In addition to setting amyloid fibrillation at low pH conditions, in order to cause partial unfolding and drive amyloid fibrillation of β_2_m at neutral pH, a plethora of conditions has been suggested, such as adding glycosaminoglycans, detergents, denaturants or by using ultrasonication and elevated temperature [53,74,75,76,77,78]. These intrinsic and extrinsic factors increase the concentration of a partially unfolded intermediate in which the natively *cis*-configured proline 32 in the polypeptide chain is isomerized to a *trans* isomer [53,79]. In addition, solid-state NMR studies have shown that amyloid fibrils which form from acid-denaturated β_2_m at pH < 3 contain a *trans* Pro 32 as well [46,80]. It should be noted that this is not the only structural change reported to be associated with amyloid formation as β-sheets in the protein turn from antiparallel in native β_2_m to parallel in the amyloid [46]. However, *cis-trans* isomerization of Pro 32 is considered as a crucial trigger for the transition of soluble monomeric β_2_m to its misfolded amyloidogenic species [7,8]. This hypothesis is supported by the observation that in the ΔN7 variant of β_2_m, where the first 7 N-terminal residues are truncated, the *cis*-Pro 32 conformer is destabilized in such a manner that only the *trans*-Pro 32 exists at neutral pH [47].

So far, it has been proven that a single region, approximately 10 residues long (60–70), is crucial for elongation of the full-length protein under certain conditions [81]. Aromatic residues are widely present in this region, which most probably contributes to the propensity of β_2_m to aggregate [70]. Studies of the full-length protein sequence at low pH have shown that shifting certain residues, especially Leu 23, His 51 and Val 82 with Pro which acts as β-sheet breaker, causes a lowering in fibril elongation kinetics. Moreover, when comparing intact protein at low pH and peptide studies in the context of the effects of sequence alteration on the fibril growth kinetics, results are surprising. Namely, isolated fragments including residues 20–40, 60–70 and ~80–99 all form amyloid fibrils [71,82], whereas in the full-length protein chain mutation of residues, only the ~60–70 region has altered fibril formation kinetics [81,83]. Results of NMR studies explain this observation; the acid-unfolded non-native structure of β_2_m is stabilized by the disulphide bond and includes gathering of hydrophobic residues in two regions (29–51 and 58–78) [8], meaning that a single strain of 10 residues might have a strong impact on the aggregation potential of the entire protein. It is speculated that this might be a result of an evolutive twist [79]. Namely, this sequence includes aromatic residues such as Phe 55, Trp 60, Phe 62, Tyr 63 and Leu 65, which are important for interaction with the MHC I heavy chain [79] and hence for regulation of immune system.

As mentioned above, the *cis* Pro 32 conformer is proposed as an essential residue for β_2_m nucleation at neutral pH and P32G and P32V mutants have been used to show this. Namely, both mutants adopt *trans* Gly or Val 32, respectively, but cannot form amyloid-like fibrils spontaneously, even though P32G can elongate preformed seeds more efficiently than wt β_2_m [8,55]. These acyclic amino acids favor the *trans* conformation at the peptide bond, but it is obvious that they cannot completely imitate the unique conformation of Pro 32 [79]. Moreover, variants such as P5G and ΔN7 also affect isomerization of the Pro 32 peptide bond, facilitating fibril nucleation at pH 7.0 [53,84]. On the other hand, β_2_m can form oligomers and fibrils at neutral pH by addition of Cu^2+^ and 1 M urea [7]. Namely, peptide bond isomerization at Pro 32 can be initiated by the coordination of a metal ion causing the rapid formation of oligomers [7,53]. Therefore, the isomerization of Pro 32 has been constantly shown as a key initial step in β_2_m amyloid fibrillation [85].

In summary, aggregation of β_2_m into amyloid structures may be achieved via a numerous routes as β_2_m forms amyloid fibrils at both pH 2.5 and 7.0 [53]. There are many avenues that might finally lead to a better understanding of the assembly pathways in different conditions. In both cases, interactions between specific hydrophobic and aromatic residues may lead to fibrillation. However, fibrils formed at neutral pH contain a highly-charged surface [86], which could be neutralized at low pH. This might explain the fact that fibrils form much more rapidly under acidic conditions and provides support for a convergent mechanism of assembly at acidic and neutral pH. Another important hallmark of amyloid fibrillation which is considered as a key to amyloid formation is the destabilization of the N-terminal region; a double variant P32G/17A which combines a trans peptide bond at Pro 32 with the destabilization of the N-terminal region forming fibrils spontaneously at pH 7 [86]. It remains to be elucidated whether the assembly pathways are similar and how they converge in the form of a common fibrillar structure.

### 3.3. Prediction of Stability of β_2_-Microglobulin and Its Proline Mutants

β_2_m stability was assessed using I-Mutant2.0 [45] upon Pro to Ser mutations in positions 5, 14, 32, 72 and 90 (numbering according the processed form of the protein—UniProt ID: P61769) and mutations P32G [53] and P32L on the primary and tertiary structures [87,88], respectively. Of note, all five Pro to Ser mutations destabilize the protein (−2.24 to −0.99 Kcal/mol) as well as the β_2_m-P32G mutation (−2.62 to −1.74 Kcal/mol) (Table 2). On the other hand, the β_2_m P32L mutant exhibits a destabilizing effect on its primary structure (−2.05 Kcal/mol) and a stabilizing effect on the tertiary structure of its monomeric [88] (1LDS:A, 0.79 Kcal/mol) and dimeric (3LOW:A [87], 1.05 Kcal/mol) forms, respectively (Table 2).

### 3.4. Structures of β_2_-Microglobulin Monomer and Domain-Swapped Dimer

**Figure 6:**
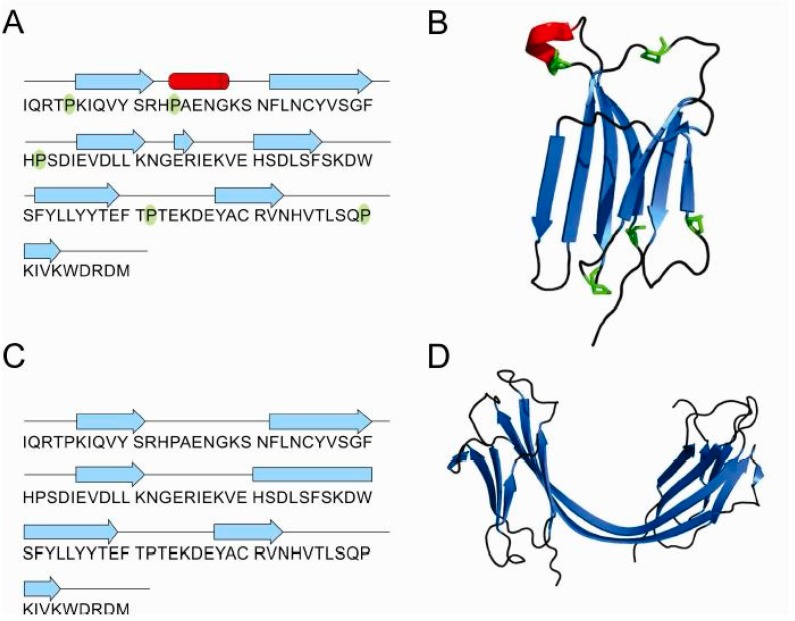
β_2_-microglobulin schematic presentation of secondary structure elements and the 3D structure of monomer (PDB id: 1LDS) [88] (**A**,**B**) and domain-swapped dimer (PDB id: 3LOW) [87] (**C**,**D**). All five prolines are highlighted in green in the monomer.

3D structures of the β_2_m monomer, dimer and tetramer are also known (Figure 6A–D). Some studies suggest that different reagents can trigger different oligomerization pathways [53]. Namely, the crystallographic structure of the reductant-triggered β_2_m dimer [87] was different from the dimer and hexamer triggered by copper [7,89] suggesting that different conditions alter the protein structure in different ways, leading to different results. Liu et al. [87] suggested that dimerization of β_2_m may occur via a relatively uncommon run-away domain swap with a covalent linkage where β strands are exchanged between two subunits, creating two interfaces. One is called the closed interface, and the other the open interface due to a new β-sheet that contributes to the stability of dimer. Another hallmark of this phenomenon is the rearrangement of the disulfide bonds as they serve as an intermolecular bond to stabilize the dimer. Moreover, in cases where natively-folded proteins form amyloids, a newly formed cross-β spine is required for the fibril ensemble and in the cases of domain-swapping, studies have shown that the hinge loop is essential for forming the cross-β spine. The LSFSKD structure (residues 53–58 of human β_2_m) acts as a typical steric zipper structure. Upon reduction of the intramolecular disulfide bond, the β_2_m monomer can assemble as “closed-ended” oligomers or “open-ended” runaway domain-swapped oligomers. The formation of intermolecular disulfide bonds stabilizes the domain-swapped oligomers. The self-association of hinge loops into a zipper spine allows the transformation from oligomers into fibrils; as the oligomer grows, the loop regions between swapped domains can slide slightly to fit into a particular frame. Based on these findings they postulated the so-called “domain-swapped zipper-spine model” of a β_2_m fibril [87].

## 4. Materials and Methods

### 4.1. Protein Isolation

In brief: the recombinant wild-type like, C3S E31-stefin B and its corresponding proline mutants were expressed in *E. coli* and purified by carboxymethylated (CM) papain Sepharose affinity chromatography. The unbound material was eluted with 0.01 M Tris-HCl containing 0.5 M NaCl at pH 8.0. Stefin B protein was eluted with 0.02 M triethanolamine (TEA) buffer at pH 10.5 and was fast refolded into a stronger buffer of a neutral pH. Furthermore, in the cold room, gel-filtration on Sephacryl S-200 was performed using phosphate 0.01 M buffer pH 7.5., 0.12 M NaCl. For analytical purposes, size-exclusion chromatography was used. Using a Superdex 75 column (Pharmacia, Uppsala, Sweden), stefin B eluted as a set of oligomers: monomers, dimers, tetramers and higher oligomers.

All other methods: expression, isolation and purification of stefin B wt and mutants as well as the conditions to follow fibril fluorescence by ThT fluorescence, were the same as previously described [20,90].

### 4.2. Fluorescence Spectra

Fluorescence was measured using a model 1.2× fluorimeter from PTI-Photon Technology International (Birmingham, NJ, USA) with a thermo unit for temperature control. 1-anilinonaphthalene-8-sulfonic acid (ANS) fluorescence was measured using an excitation wavelength of 370 nm and spectra were recorded from 400 to 600 nm. The entrance and exit slits for the excitation light-beam were 3 nm, 2 nm and 2 nm, respectively. Measurements were made in a 10-mm micro-cuvette at 25 °C. Thioflavin T (ThT) fluorescence was measured using an excitation wavelength of 440 nm and emission wavelength of 482 nm.

### 4.3. Circular Dicroism Specta

CD spectra were measured using an Aviv model 62A DS CD spectropolarimeter (AVIV, Lakewood, NJ, USA). Far-UV CD spectra were recorded in a 0.1 cm cell. Protein concentration was 34 μM or lower for the far-UV CD. For the far UV CD the bandwidth was 1 nm, and the step of measurement was 1 nm, with data integration time 4 s. Measurements were performed at 25 °C.

### 4.4. Size-Exclusion Chromatography (SEC)

The oligomeric state and purity of the protein samples was determined by size-exclusion chromatography (SEC) using a Superdex 75 FPLC column (Pharmacia, Uppsala, Sweden). The flow rate was 0.5 mL/min and typically a 100 μL of 50 μM sample of the protein was applied. Buffer was 10 mM potassium phosphate, pH 7.0, with 0.15 M NaCl added—if not otherwise specified.

## 5. Conclusions

Studies on both stefin B and β_2_m indicate that there is a link between oligomerization and *cis* to *trans* isomerization of certain Pro residues. For β_2_m, in the monomer, Pro 32 is found in a *cis* conformation. In this case, *cis* to *trans* isomerism leads directly to fibril formation, whereas in stefin B the *trans* to *cis* isomerization leads to the off-pathway tetramer [91] so that yet another transition from *cis* to *trans* is needed for fibril elongation [91]. In conclusion, *cis* to *trans* isomerization of a critical proline may act as a switch towards amyloid fibrils, starting with domain-swapping. Neighboring residues of the proline undergoing *cis/trans* isomerism are also important for the regulatory switch, such as lysine or serine/threonine residues, in prion and phosphorylated Tau, respectively [92,93].

## Figures and Tables

**Table 1:** Prediction of human stefin B stability upon proline mutations.

Mutation	ΔΔGP04080|C3S (Kcal/mol)	ΔΔG1STF:I (Kcal/mol)	ΔΔG4N6V:0 (Kcal/mol)	ΔΔG2OCT:A (Kcal/mol)
P6L	1.23	P11L 1.47	NA	1.17
P11S	−0.71	P16S -0.20	−0.30	−0.42
P36D	−0.89	P43D -0.83	−0.69	−0.69
P74S	−1.64	P103S -0.82	−1.44	−0.48
P79S	−1.88	P107S -0.67	−1.19	-

The predicted change in stability of human stefin B (P04080|C3S) upon single-point proline mutations was performed at pH 7.0 and 25 °C using I-Mutant2.0 software [45]. The free energy change of protein stability (ΔΔG) is the difference between the ΔG_wild-type_ and the ΔG_mutant_ expressed in Kcal/mol. ΔΔG < 0 indicates a destabilization of the protein upon mutation (a higher negative value of the ΔG_wild-type_), whereas a ΔΔG > 0 indicates an increase in mutant’s stability (a higher negative value of ΔG_mutant_). In the 3D structure 1STF:I [36], the labeling of the amino acid residues differs from the wild-type protein, therefore, its numbering was indicated accordingly. The monomeric 3D structure 4N6V [44] lacks the first seven amino acid residues, thus including Pro 6; therefore, this value is missing in Table 1 (NA—not available). In addition, in the 3D structure 2OCT [19], the residue at position 79 was already serine; therefore, no prediction was done in this case (-).

**Table 2:** Prediction of stability of human β_2_-microglobulin proline mutations.

Mutation	ΔΔGB2MG|21-119 (Kcal/mol)	ΔΔG1LDS:A (Kcal/mol)	ΔΔG3LOW:A (Kcal/mol)
P5S	−0.99	−2.50	−1.93
P14S	−1.85	−0.55	−0.94
P32G *	−2.62	−2.15	−1.74
P32L *	−2.05	0.79	1.05
P32S	−2.24	−0.90	−0.57
P72S	−1.74	−0.61	−0.81
P90S	−1.28	−1.92	−1.81

The predicted stability of β_2_-microglobulin (P61769|21-119) and its single-point proline mutants, some of which were used for folding studies (*), was done at pH 7.0 and 25 °C using I-Mutant2.0 software [45]. The free energy change of protein stability (ΔΔG) is the difference between the ΔG_wild-type_ and the ΔG_mutant_ expressed in Kcal/mol. A ΔΔG < 0 indicates a decrease in protein stability whereas a ΔΔG > 0 indicates a stabilization of the protein.

## Supplementary Materials

Supplementary materials can be found at www.mdpi.com/1422-0067/18/3/549/s1.
