# Supplementary material for: Proline Residues as Switches in Conformational Changes Leading to Amyloid Fibril Formation
Source: Int J Mol Sci. 2017 Mar 7;18(3):549. doi: 10.3390/ijms18030549 (PMC5372565; doi:10.3390/ijms18030549)
Supplement: Supplementary file 1 [file ijms-18-00549-s001.pdf]

Supplementary Materials: Proline Residues as Switches in Conformational Changes Leading to Amyloid Fibril Formation

Ajda Taler-Verčič, Samra Hasanbašić, Selma Berbić, Veronika Stoka, Dušan Turk and Eva Žerovnik

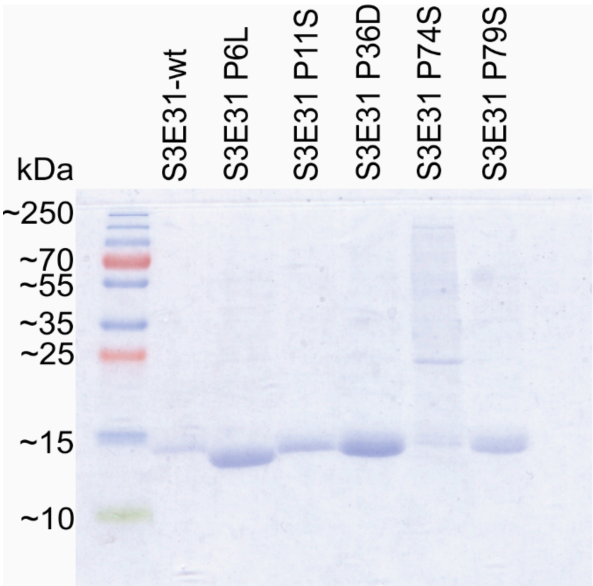

Figure S1. SDS PAGE with wt stefin B and all proline mutants.

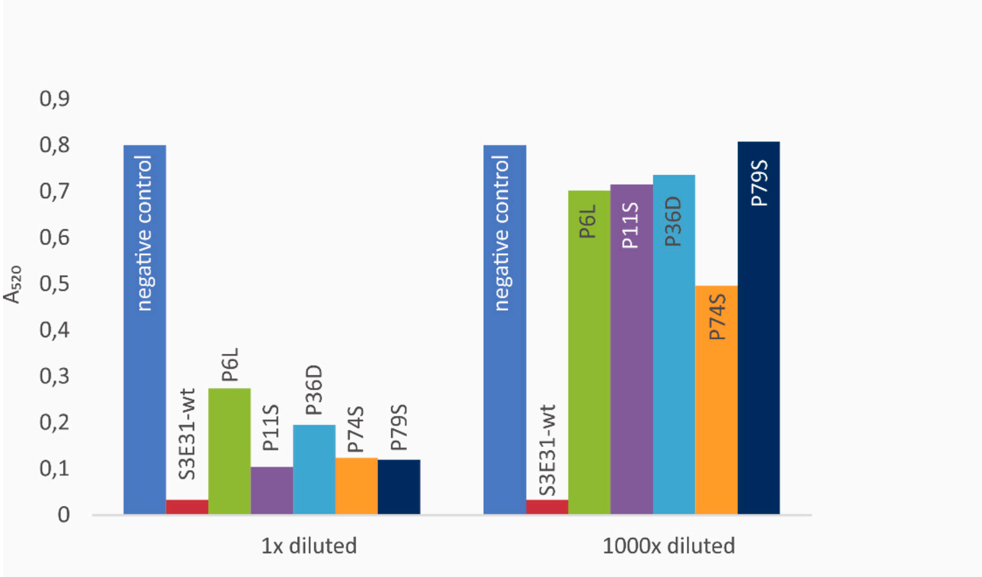

Figure S2. BANA test for protein inhibitory activity. Higher values present no inhibitory activity. Bacterial lysates after production of recombinant proteins were used. All proteins (wt and mutants) were active and the activity was no longer detected after sample dilution.

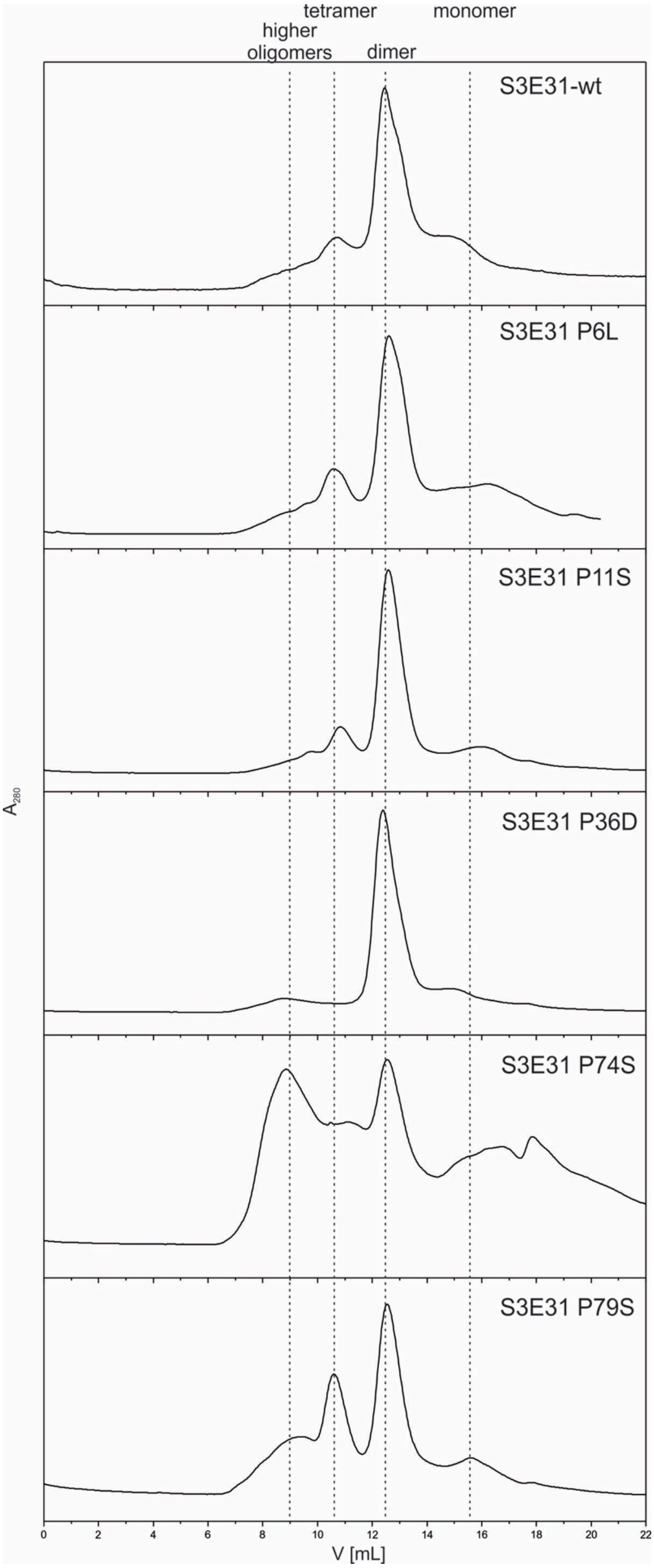

Figure S3. SEC profile of the wt protein and all five mutants. A Superdex 75 10/300 column was used on an FPLC system at room temperature. The column was equilibrated with 0.01 M phosphate buffer, pH 7, and 0.15 M NaCl. Dashed line indicates the area where certain oligomeric species elute from the column.
